# A Bayesian Framework for the Automated Online Assessment of Sensor Data Quality

**DOI:** 10.3390/s120709476

**Published:** 2012-07-11

**Authors:** Daniel Smith, Greg Timms, Paulo De Souza, Claire D'Este

**Affiliations:** 1 Intelligent Sensing and System Laboratory (ISSL), Commonwealth Science and Industrial Research Organisation (CSIRO), CSIRO Marine and Atmospheric Laboratories, Castray Esplanade, Hobart 7001, Australia; 2 Human Interface Technology Laboratory, University of Tasmania, Launceston 7250, Australia

**Keywords:** online filtering, automated, quality assessment, sensors, dynamic Bayesian networks

## Abstract

Online automated quality assessment is critical to determine a sensor's fitness for purpose in real-time applications. A Dynamic Bayesian Network (DBN) framework is proposed to produce probabilistic quality assessments and represent the uncertainty of sequentially correlated sensor readings. This is a novel framework to represent the causes, quality state and observed effects of individual sensor errors without imposing any constraints upon the physical deployment or measured phenomenon. It represents the casual relationship between quality tests and combines them in a way to generate uncertainty estimates of samples. The DBN was implemented for a particular marine deployment of temperature and conductivity sensors in Hobart, Australia. The DBN was shown to offer a substantial average improvement (34%) in replicating the error bars that were generated by experts when compared to a fuzzy logic approach.

## 1. Introduction

Moore's Law is primarily responsible for bringing to fruition the technical advances in sensor development that have led to the explosion in scientific data being generated each year [1]. Furthermore, the utilisation of digital technologies, in conjunction with the Internet, has altered the traditional role of data stewardship. We are now moving into an age where data storage is distributed and data is publicly accessible and annotated to promote re-use for a variety of purpose. Consequently, the explosion of open data provides the opportunity to study the environment at a higher spatiotemporal resolution than was previously possible. Observation scale is only one of the critical aspects needed to advance our understanding of the physical world. The integrity of these observations must also be assessed to ensure that they are fit for purpose [1]; this becomes increasingly challenging as the size and heterogeneity of data sets used in scientific studies continue to expand. Automated approaches to assess the quality of data are important as the number of observations being generated make human based assessment too unwieldily and costly [1,2]. Furthermore, automated procedures are essential to perform quality assessments for real time applications that impose tight time constraints upon processing [3–5].

One of the issues that is critical to assessing the quality of sensor data is contextual awareness. For instance, context can provide a means to differentiate between a sensor fault and an unusual, real event in the environment. In this case, exploiting our understanding of the sensed phenomenon and the structure of associated events [6] or learning the spatiotemporal relationship between a sensor and its neighboring sensors [7] can assist in resolving this ambiguity [5].

One of the weaknesses of automatic quality algorithms is that they do not utilise context as effectively as a human expert [8]. Commonly, evidence streams (the quality tests) of automatic quality assessments are independently processed and then combined into an assessment using logic based operators. Furthermore, the tests associated with current approaches are commonly represented as deterministic variables that do not represent the sources of uncertainty associated with each of the quality tests. We propose a framework to address these two issues for online automated assessment of sensor data. The probabilistic framework is based upon the Bayesian networks (BN), a directed acyclic graph that can explicitly model the dependencies between random variables, making its usage attractive to sensor data fusion research [9–11]. Dynamic Bayesian Networks (DBN) have previously been proposed to detect sensor reading outliers by identifying significant predictions errors made by Kalman and particle models [4] or the Hidden Markov Model [7] based upon spatiotemporal redundancies. In contrast, our proposed approach is not a prediction model but a framework to represent and combine the sources of uncertainty associated with the data quality of a sensor. This framework can represent any set of quality tests and assessment format. Context can be modeled by representing the causal relationship between tests. Another novel aspect of this approach is the manner in which probabilistic assessments are combined to produce a measure of sample uncertainty. This proposed quality measure is different from previous work with BNs that calculate error bars for the posterior of each state query using a first order Taylor expansion [12] or two independent sets of network parameters [13] to approximate the variance of the posterior distribution. For quality control problems, error bar approaches can be used to compute the uncertainty of each quality state query but do not provide an overall estimate of the sample uncertainty. Our proposed approach provides an uncertainty estimate of each sample by combining the weighted posterior probabilities of the quality states. It is similar to the metric used with fuzzy logic sets [14] where the proposed uncertainty measure was shown to reproduce the error bars generated by an expert. This quality assessment framework was first proposed as a static BN that was best suited to assessing the data quality of stochastic time series [15]. The observations of most phenomenon are sequentially correlated over short periods of time. Consequently, the original framework is reformulated as a Dynamic Bayesian Network (DBN) [16] to provide online quality assessment of sensor readings that are correlated across time. The framework was implemented by developing a separate DBN to assess the data quality of each conductivity and temperature sensor deployed in the Derwent estuary in Hobart, Australia. The quality assessments of the DBN were compared to the equivalent static BN with respect to three months of sensor data that was assessed by quality control experts. The sample uncertainty measure of the DBN were then compared to a similar measure from the fuzzy logic approach [14] and expert generated error bars.

## 2. Previous Work

Previous approaches to automate the quality assessment of sensor data can be broadly categorized into two classes. The first class are anomaly detection methods; these approaches do not provide explicit classification but perform the related task of detecting samples that deviate from expected behavior [4,7,17–19] and then flagging these as outliers. These are considered bottom-up, statistical approaches that are trained from historical data sets of the sensor. The second are classification approaches that use prior knowledge to label the quality of sensor data [14,20–23]. Our proposed DBN is a combination of both approaches. Networks are trained from historical data sets that can be contextualized by encoding prior knowledge about sensor operation and its measured phenomenon. In Sections 2.1 and 2.2 we introduce anomaly detection and classification approaches, and specify the potential advantages that the Bayesian framework offers over each approach individually.

### 2.1. Anomaly Detection

Anomaly detection methods are statistical approaches that can exploit historical distributions of sensor behavior. In anomaly detection, models are typically used to predict the behavior of sensor readings based upon their temporal correlation [17,24] or spatiotemporal correlation [4,7,18,19] with neighboring sensors; samples with values that deviate significantly (usually defined by a threshold) from the predicted value are flagged as outliers. The advantage of anomaly detection methods is that they have broad application, given that the assessment procedure can be treated as a black box. This is also a weakness when attempting to detect systematic errors. The detection of systematic errors is assisted by having some understanding of the sensor pathology and its measured phenomenon [5]. Our framework can address this issue by incorporating prior knowledge of the quality tests into the DBN.

Another issue with anomaly detection methods is that they rely upon assumptions of spatial, temporal or spatiotemporal correlation that are not universally applicable to particular types of phenomenon [25]. Such correlation assumptions rely upon data following the same distribution. However, this does not hold across all types of networks. For instance, it does not hold for networks comprised of acoustics or thermal sensors with readings that attenuate with respect to the source distance [26]. More importantly, the anomaly detection methods require sensors to be deployed at a sufficiently high spatial resolution to adhere to the spatial correlation assumptions. Networks with a sufficiently high spatial density may be financially, logistically or technically prohibitive to deploy in certain scenarios, meaning that alternate quality assessment approaches may need to be considered. The proposed framework has more generic application than previous anomaly detection work, as any quality test or assessment format can be incorporated into the framework. This is beneficial as it allows quality assessments to be tailored to particular sensors based upon their physical location and function. Moreover, it allows assessments to be fit to standard procedures for a particular domain. New quality tests can be added or removed from an existing BN without requiring the entire network to be retrained.

### 2.2. Classification Based Assessments

Classification based assessment are top-down approaches that exploit knowledge of the sensed phenomena in order to infer the quality state. Consequently, classification approaches have a far stronger connection to their application domain; this is often achieved by establishing a series of rules. For instance, a series of thresholds were obtained from scientists to form data quality rules for a soil moisture network [21]. Knowledge of specific systems can also be used to validate sensors and detects faults in engineered systems including the power system of aerospace vehicles [27], robotic vehicles [28] and gas turbines [29]. A network is used to model the the overall system, in particular the functional dependencies between the states of its sensors. Such systems model the uncertainty of sensor operation but not the uncertainty associated with the system process given the narrow bounds of its behaviour. Environmental process are far more complex to model given the variety of contributing factors and fluctuation with location. To assess the data quality of environmental sensors, this process uncertainty needs to be represented in the network. Consequently, our proposed framework models both the process and operational uncertainties of each sensor individually. Given our current focus on marine applications, the rest of this section is spent reviewing notable examples of automated quality procedures for operational marine observation networks. The Argo float project has deployed over 3,000 profiling floats throughout the world's oceans and performed automated, real-time quality assessment of data as a part of a more extensive Quality Assurance (QA) and Quality Control (QC) process. A set of automated tests including spike, gradient, regional range, pressure increase and density inversion have been applied to the temperature, salinity and pressure series [22]. Koziana described the QA/QC framework and the use of measurement range and gradient data checks as part of the U.S. Integrated Ocean Observing System (IOOS) [30]. The National Reference Stations (NRS), part of the more extensive Integrated Marine Observing System (IMOS), is a group of nine coastal monitoring stations distributed along Australia's coastline. An automated procedure for assessing the quality of temperature and salinity data is currently being developed by deploying gradient, spike and climatologic distribution tests [23]. In each of these quality assessment systems, “gold standards” (*i.e.*, thresholds) are defined for each of the quality tests in order to indicate whether the test has been passed. When a data sample fails a test, it is flagged as “bad”. Such approaches fail to exploit the contextual relationship between different quality tests and test uncertainty. The proposed BN framework explicitly models the causal relationships between tests providing additional context to the quality assessment. Furthermore, the BN provides probabilistic assessments that provide a natural way to represent the uncertainty associated with imperfections in the quality tests. We argue that such an uncertainty measure provides a more valuable criteria than a discrete data flag (such as “poor” and “good”) in order to assess a sample's fitness for purpose.

Timms [14] addressed this issue of sample uncertainty by utilizing fuzzy logic to represent the uncertainties associated with each of the assessment tests. Assessments were generated by combining tests to produce a continuous quality scale to estimate data quality uncertainty. Although our framework has been inspired by Timms's approach to combine the test uncertainties, the use of a BN can potentially provide the following benefits:

1. Network parameters are learnt via supervised training of the sensor data; the fuzzy functions in [14] were parameterized with prior knowledge that may not be representative of a particular site;
2. The network parameters can be updated if assessments of some new sensor readings are obtained;
3. The dependencies between the quality tests can be explicitly encoded, whilst tests in the fuzzy system are independent. This provides additional context and makes the fuzzy system more prone to failure when particular evidence sources are unavailable.

This fuzzy system is compared to the Bayesian Network systems in Section 7.3.

## 3. Bayesian Networks

**Figure 1.**
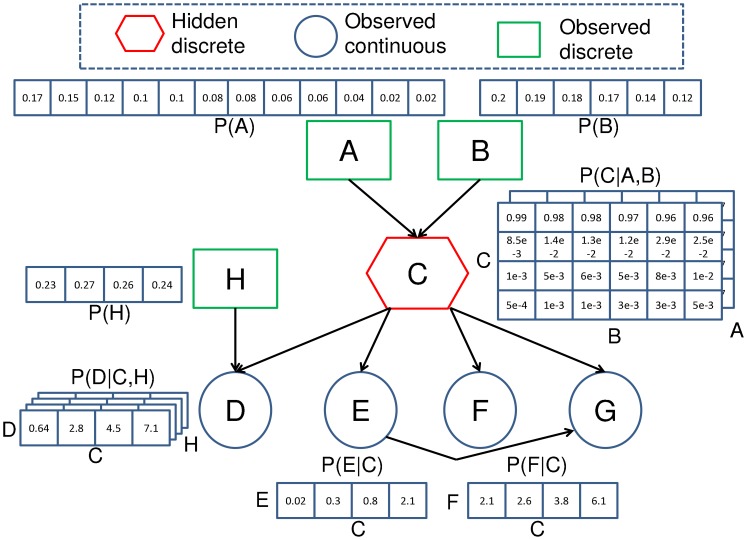
A Bayesian network is a directed acyclic graph representing the joint probability of a problem. Each node in the network is associated with a conditional probability distribution of a variable that is conditioned upon other variables with edges pointing towards it. This particular network structure is used to assess the data quality of temperature and conductivity sensors deployed in Sullivans Cove, Hobart with cause and observed evidence tests. The causes of sensor degradation include the time since the sensor was calibrated (node **A**) and the time since the sensor was cleaned (node **B**). Node **C** was used to infer the latent quality state. The observed evidence of the data quality was the seasonal difference (node **D**), the gradient (node **E**), the difference between the sensor and hydrodynamic model (node **F**) and the difference between equivalent sensors at alternate depths (node **G**). The CPD of the network have been trained from observations of a temperature sensor deployed at 1m in Sullivans Cove.

A Bayesian Network (BN) is a model representing the joint probability of a process or problem via a directed acyclic graph. The graph represents the joint probability via a number of explanatory random variables and their associated statistical relationships. In Figure 1, the BN is a joint probability distribution that is used to provide quality assessments of temperature sensor data. The cause and effect tests associated with the network are represented by a set of discrete and continuous variables *X* = [*x_A_*, *x_B_*…x*_H_*]. Each of the variables in the network are associated with a node (different shapes in Figure 1) representing the conditional probability distribution (CPD) of the variable that is conditioned upon other variables with edges pointing towards it. The nodes that point towards a particular node are known as its parents.

One of the basic properties of a BN is that it satisfies the local Markov property that states a node is conditionally independent of non-descendant nodes in the network given its parents [31]. Using the chain rule and this local Markov property, the joint probability of a BN *p*(*X*) can be represented as the product of a number of conditional probability distributions:

(1) $$p(X)=\prod_{i=A}^{H}p(x_{i}|pa_{i})$$

where each node is represented by a probability distribution that is only conditioned upon its parent *pa_i_* nodes *i.e.*, *p*(*x_i_*\*pa_i_*). Whilst the original joint distribution *p*(*X*) of *N* discrete variables (each with *k* values) exhibits a combinatorial explosion in the number of potential states (*i.e.*, O(*k^N^*)), this is significantly reduced in a BN by exploiting the conditional independence of the variables [32]. The construction of a BN relies upon prior knowledge of the system being modeled. For instance, the network designer must be able to determine which variables contribute to the system, how the variables should be represented and which variables are conditionally dependent upon one another. Although automated approaches to learn network structure have been developed [33,34], human based specification of the network is likely to be more reliable for a well-understood problem such as data quality.

## 4. Bayesian Network Framework for Quality Assessment

**Figure 2.**
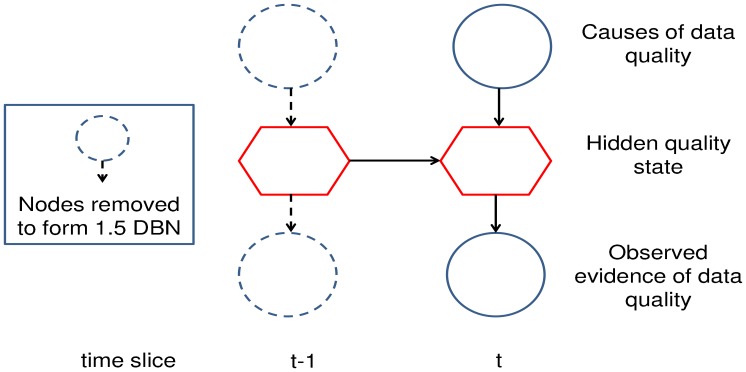
The two time-slice structure of the Dynamic Bayesian Network used to perform quality state inference for each incoming sample. The interface algorithm only requires the node variables from the previous slice that are connected to the current slice to be involved in the quality state inference. This is known as the 1.5 DBN structure.

The proposed framework for online, automated quality assessment of sensor data uses a BN to represent the cause and effects of data quality in consecutive layers of the network. This framework is shown in Figure 2. The top layer of the network represents the observed causes of sensor data quality. These variables can represent any number of causes of degradation in observations including connection or hardware failure [8,21,35], clipping due to insufficient dynamic range in the analogue to digital conversion [8], low battery levels [21,35], sensors with calibration drift [8,21] and sensors that have been bio-fouled [36] in their deployed environment.

The middle layer of the network represents the latent quality state of the sensor. This quality state is inferred from the cause and observed evidence variables above and below it in the network. The quality states of the network depend upon the particular quality assessment scheme that has been adopted for the application. The bottom layer of the network represents the sources of evidence used to infer the quality of the current sample. The evidence observations for an assessed sensor are commonly the sources of spatial, temporal and/or seasonal redundancy that can be used to detect contradictory behavior associated with errors in the sensor readings.

### 4.1. Dynamic Network for Quality Assessment

Sensors quite often measure environmental phenomena that are correlated across time. For such phenomena, there will be dependencies between the current quality state and the quality state at previous time steps. The DBN framework shown in Figure 2 adopts the simplest temporal model, a first-order Markov model, where the dependencies between quality states are only modeled between consecutive time steps. When each of the observed variables is singly connected to the quality state, the network can be considered to be a special variant of the Hidden Markov Model (HMM), known as the Input-Output HMM [37]. The hidden state CPD of the standard HMM has static state transition probabilities (*i.e.*, *p*(*quality_t_*|*quality_t_*_−1_)), whilst the Input-Output HMM has state transition probabilities with greater dynamics given that they are also dependent upon the causes of data degradation (*i.e.*, *p*(*quality_t_*|*quality_t_*_−1_, *causes_t_*)).

### 4.2. Online Quality Inference

Online quality inference of an incoming sample can be performed by filtering across a sequence of past observed variables and quality states:

(2) $$p(q(t)|E(1:t),q(t-1))=\frac{p(q(t)|q(t-1))\cdot p(q(t)|E(t))\cdot p(q(t-1)|E(1:t-1))}{p(E(t)|E(1:t-1))}$$

where *E*(1 : t) represents the observed effect and cause variables of the DBN up to the current time t. Filtering is performed with the interface algorithm [33] using the feed forward algorithm in the form of Equation (2).

The interface algorithm performs a modified junction tree inference upon a 2 slice network comprised of consecutive time slices *t* − 1 and *t*, given past time slices are independent of future time slices given the current interface. Infact, only nodes in slice *t* − 1 with children in slice *t* need to be part of the inference. This is known as a 1.5 slice DBN and is shown in Figure 2.

The number of operations required to perform an inference for each time step is bounded between *O*(*K^I^*^+1^) and *O*(*K^I^*^+^*^N^*) where *N* is the number of hidden state variables in the current time slice, I is the number of variables in the forward interface (number of variables with children in the next time slice) and *K* is the number of states per hidden variable [33]. The complexity of this inference would be computationally prohibitive in a network where the state space was comprised of a significant number of variables dependent upon the next time step. The interface algorithm was suitable for our proposed framework as the quality inference in the current time step was only dependent upon the quality state of the previous time step. If the framework was modified so that variables in the current time slice had numerous parents in the previous time slice, the interface algorithm would be computationally infeasible. Exact inference may still be possible, however, the frontier algorithm [33] would be a better option. The minimal complexity of the frontier algorithm is *O*(*NK^N^*^+1^) per time step, and hence, it is computationally feasible as long as the hidden state space remains low. If this is not the case, an approximate inference engine might be necessary [33].

## 5. Tasmanian Marine Analysis Network (TasMAN)

The Tasmanian Marine Analysis Network (TasMAN) has been developed to assist with multi-use management of estuaries and coastline in South-eastern Tasmania. TasMAN is an end to end system that consists of a marine sensor network, a real-time information system and visualization tools. The system is designed to monitor the Huon and Derwent estuaries and provide alerts and forecasts. The system has also been developed as a low cost, relocatable solution for estuarine monitoring; the reuse of the TasMAN platform has been demonstrated during its deployment in Sydney Harbour and the Brisbane River [38].

The current network in Tasmania consists of fixed sensor nodes and mobile nodes including a small AUV [39] and an autonomous catamaran. The fixed nodes consist of a string of temperature, conductivity and pressure sensors that can operate as either a node within a wireless sensor network, or independently, via 3G mobile telephony. These nodes have been designed to be inexpensive and flexible to interface with a variety of different sensors. Although sensors are a variable expense, sensors of low cost such as the DS28EA00 temperature sensor, Odyssey conductivity sensor and SSI MediaSensor pressure sensor have been used in the network [38]. The BN framework for quality assessment has been developed to address some of the challenging requirements of assessing the quality of sensor data across a low cost, wide area network such as TasMAN. The benefits for deploying this quality assessment framework within TasMAN include:

1. Its flexibility and re-use. Different tests and sources of evidence can be used to generate quality assessments based upon the requirements of each sensor and the resources available at different deployment locations.
2. Quality assessments can be generated upon recently deployed sensors. The ever growing nature of our network in South Eastern Tasmania means assessments need to be generated during initial deployment periods whilst statistics are still being generated. Bayesian approaches are beneficial to regulate the network parameters when training data is limited and can be learnt sequentially to enable the update of parameters when new data from the network is manually assessed.
3. Probability based assessments can be used to quantify the uncertainty of sensor measurements. This is important given the quality of our sensors is relatively low in comparison to the expensive, high precision sensors commonly used by scientists and physical modelers.

## 6. TasMAN Quality Assessment

**Figure 3.**
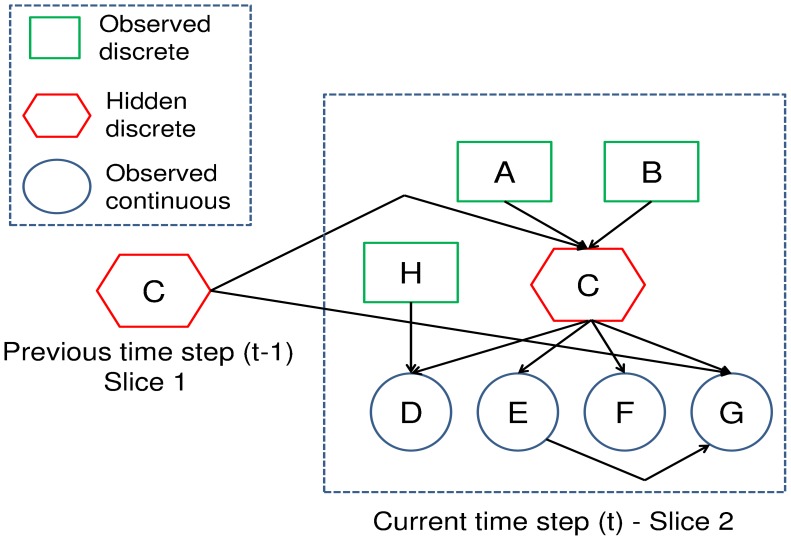
The 1.5 DBN model used to assess the data quality of individual temperature and conductivity sensors in Sullivans Cove, Hobart at 1 m and 10 m. The causes of sensor degradation in this model were the time since the sensor was calibrated in node **A** and the time since the sensor was cleaned in node **B**. The latent states used to infer the data quality in node **C** were defined by the IOC flagging standard. The observed evidence of data quality was the seasonal difference in node **D**, the gradient in node **E**, the difference between the sensor and hydrodynamic model in node **F** (only for sensors at 1 m) and in node **G** the difference between equivalent sensors at alternate depths.

A separate DBN was developed for each of the four EC-1500 temperature and conductivity sensors fixed to a wharf in Sullivans Cove, Hobart (−42.886, 147.337) at 1 m and 10 m below chart datum. The 1.5 slice DBN (shown in Figure 3) was used to perform a data quality inference upon a sensor.

### 6.1. Hidden Quality State

The quality states of this deployment are adopted from the flagging scheme used by the Intergovernmental Oceanographic Commission (IOC) of UNESCO [40]. The flags are numerical codes used to provide standard definitions of processing tasks and data quality of each measured sample. There are flags associated with particular processing tasks, *i.e.*, if a quality assessment or interpolation was performed. Four IOC flags shown in Table 1 describe discrete levels of data quality that represent the quality states of our network. The CPD of the hidden quality state *q*(*t*) (in node C of Figure 3) was discrete and conditioned upon the observed causes of the data quality and the previous quality state *p*(*q*(*t*)|*q*(*t* − 1), *cl*(*t*), *cal*(*t*)).

### 6.2. Causes

Two common causes of sensor degradation were incorporated into the DBN. The first variable was related to sensor calibration. Sensors often exhibit systematic errors in measurement accuracy that can be corrected for by performing a calibration. The calibration maps the sensor output to its expected measurement acquired from an additional sensor of known accuracy. With time, however, there is a drift in the accuracy of the calibration, which is related to the age and quality of the sensor. Consequently, sensors must be regularly calibrated according to the schedule outlined by the manufacturer. An increase in the time since the sensor was last calibrated, particularly after exceeding the manufacturers specification, was associated with additional uncertainty. Consequently, the number of days since the sensor was last calibrated *cal* was represented as a multinomial distribution (node A in Figure 3) with a bi-monthly resolution for the temperature and conductivity based DBN.

**Figure 4.**
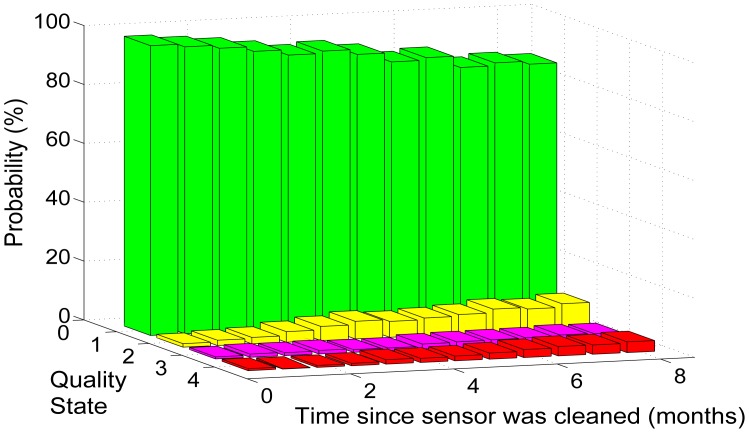
The trained CPD of the quality state conditioned upon the time since the conductivity sensor at 1 m was cleaned. The CPD was trained from the data set specified in Section 6.4 and the labels 1–4 correspond to the quality states in Table 1.

The second variable was related to bio-fouling, which is the major cause of sensor degradation in shallow marine environments. Bio-fouling of sensors in shallow seawater can occur very rapidly and lead to random fluctuations or offsets in data in less than two weeks [36]. Bio-fouling has been found to produce drifts in the type of conductivity sensors that were used in Sullivans Cove [36]. A general rule of bio-fouling drift is that, the longer it has been since the sensor was cleaned, the greater the uncertainty associated with its measurements. Consequently, the number of days since the sensor was last cleaned cl was represented as a multinomial distribution (node B in Figure 3) with a monthly resolution for both the temperature and conductivity based DBN. Figure 4 is the CPD of the conductivity sensor at 1 m depth with the quality states conditioned upon the time since the sensor was cleaned *p*(*q*(*t*)|*cl*(*t*)). The CPD was trained with the data set specified in Section 6.4. It can be seen there is a trend of the probabilities associated with the “degraded” quality states increasing and the “good” quality class decreasing as the time since the sensor was last cleaned extends.

### 6.3. Observed Evidence

Different sources of temporal and spatial redundancy were used to model sensor readings and train the DBN. The DBN was then used to infer the quality state of the incoming sample. Two forms of temporal redundancy were used to model the current temperature and conductivity readings: firstly, the short-term correlation associated with samples acquired from the sensor's recent past, and secondly, the seasonal trends acquired from far longer historical records of the sensor. In addition, two forms of spatial redundancy were used to model the current sample of each sensor. This included the gridded hind casts from the Sparse Hydrodynamic Ocean Code (SHOC) model in Sullivans Cove [41] and the readings from a co-situated sensor in Sullivans Cove at a different depth within the water column.

#### 6.3.1. Seasonal Range Test

The first test involved computing seasonal distributions for each of the four sensors. The absolute difference *x_sd_*(*t*) between each sensor reading *X*(*t*) and its corresponding seasonal distribution was then computed:

(3) $$x_{sd}(t)=|\frac{X(t)-\mu_{s}}{\sigma_{s}}|$$

where *μ_s_* is the mean and *σ_s_* is the standard deviation of the normal distribution of the season s = [winter,spring,summer,autumn]. The CPD in node *D* was parameterized from the training data set as a Gaussian distribution of the normalized seasonal difference conditioned upon the hidden quality state and season*p*(*x_sd_*(*t*)|*q*(*t*), *s*(*t*)).

#### 6.3.2. Short Term Temporal Evidence

The second test used a gradient filter to identify sudden changes in the readings between consecutive samples:

(4) $$x_{gr}(t)=|\frac{dX}{dt}|=|\frac{X(t)-X(t-1)}{\Delta t}|$$

where Δt is the time between consecutive samples. Significant gradient values are often associated with a salient change in the statistics of the measured phenomenon that are indicative of a change in its behavior. The CPD of node *E* is a Gaussian distribution of the gradient conditioned upon the four hidden quality states *p*(*x_gr_*(*t*)|*q*(*t*)). Spike detection is not incorporated within this suite of tests as it requires the use of a future sample, and hence, introduces a one sample delay into the data quality inference. Such a delay may be tolerated in messaging systems as long as the sensor's sampling rate is sufficiently high.

#### 6.3.3. Evidence from Model Hindcasts

This third test exploits spatial redundancy by training the networks of the shallow sensors with the differences between sensor readings and their corresponding model hindcasts in Sullivans Cove. The model hindcasts were provided by the SHOC model that operates within the Derwent Estuary. It produced three-dimensional distributions of temperature, salinity, current velocity, density, passive tracers, mixing coefficients and sea level upon a grid that was non-uniform and curvilinear with grid spacings of between approximately 200 m and 800 m [41]. Only the shallow pair of sensors in Sullivans Cove (at depths of 1 m) included model hindcasts in their DBN. This is because the SHOC model provided hindcasts at a depth of 1 m every 30 minutes whilst the hindcasts at a depth of 10 m were only obtained every 12 hours. Consequently, the current model did not provide sufficient temporal resolution to compare to the sensors at 10 m that were acquiring readings every 10 minutes.

The readings from the conductivity sensor were converted to salinity [42] to enable direct comparison with the salinity hindcasts from the SHOC model. It was important to note that model hindcasts and sensor readings were not guaranteed to be sufficiently correlated to use as a quality test, particularly given the uncertainty involved in comparing these two data sources (the potential sources of uncertainty are the model hindcast itself, the sensor reading conversion from conductivity to salinity and the spatial uncertainty involved in comparing a sensor at a particular point to a grid cell with coarser resolution between 200 m and 800 m). Consequently, a correlation analysis was performed upon the training set to validate the relationship between the model and sensor prior to incorporating the test into the network. It was found that the sensors and their corresponding SHOC hindcasts had Pearson correlation coefficients of 0.76 and 0.69, respectively. Consequently, there was sufficient redundancy to incorporate this test within the network given the test uncertainty could be represented by the DBN. A linear regression model of the sensor data and corresponding SHOC hindcasts in Sullivans Cove was computed from the training data set as follows:

(5) $$\begin{array}{cc} {\text{arg min}}_{\beta ,C} & \sum_{t=1}^{N}x_{mr}{\left(t\right)}^{2}\\ x_{mr}(t) & =\left|X(t)-\hat{X}(t)\right|\\ \hat{X}(t) & =\beta \cdot M(t)+C \end{array}$$

The CPD in node *F* was a Gaussian distribution of the absolute error between the model and sensor *x_mr_*(*t*) conditioned upon the four quality states *p*(*x_mr_*(*t*)|*q*(*t*)).

#### 6.3.4. Evidence between Co-Situated Correlated Sensors

This test involved exploiting the spatial redundancy between pairs of co-situated sensors measuring equivalent phenomenon at 1 m and 10 m in Sullivans Cove using the training set. To verify that pairs of sensors were spatially correlated, a statistical analysis was performed upon the training set. It was found that the temperature and conductivity sensors were highly correlated at Sullivans Cove with Pearson correlation coefficients of 0.97 and 0.92, respectively. In fact, the sensor observations at 1 m and 10 m were often similar in value, indicating that Sullivans Cove was well mixed at these depths. It should be noted that this test did not require the water column to be well mixed in order to exploit it for quality assessment purposes; the test could also be used in a stratified water column if pairs of sensors were situated on the same side of the pycnocline.

**Figure 5.**
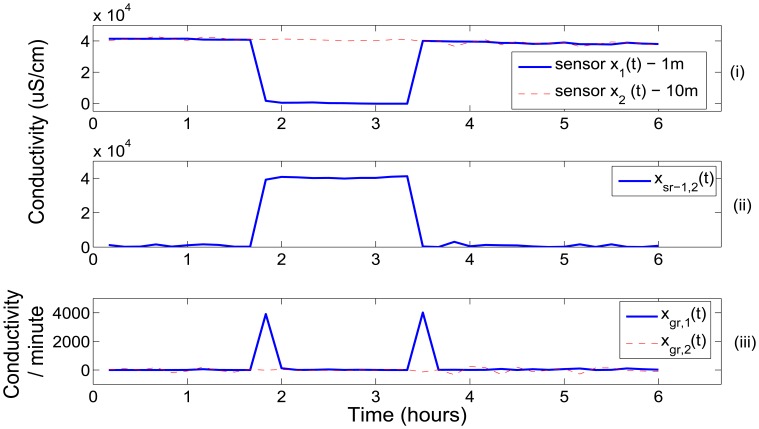
A comparison of the corresponding readings from co-situated conductivity sensors at 1 m (*x*_1_(*t*)) and 10 m (*x*_2_(*t*)). (i) The sensor *x*_1_(*t*) had a step change in its readings as a result of an electronic fault; (ii) The difference test (*x_sr_*(*t*)) between the sensors could identify the fault but can not resolve which of the sensors was responsible. The *x_sr_*(*t*) test was conditioned upon the gradient test *x_gr_*(*t*) shown in (iii) and previous class state *q_t_*_−1_ to infer *x*_1_(*t*) was erroneous.

The relationship between pairs of sensors were modeled via linear regression similarly to Equation (5), except that the hindcast *M*(*t*) was now replaced by a co-situated sensor. High errors between the current assessed sensor and the regression forecast were indicative of potential faults in sensor readings. However, the test did not resolve which sensor in the linear regression model was erroneous. This can often be resolved by conditioning the regression error *x_sr_*(*t*) upon the gradient *x_gr_*(*t*) of the assessed sensor. Larger values of *x_gr_*(*t*) represent sudden changes in sensor readings that are responsible for the increased modeling errors *x_sr_*(*t*). The gradient, however, cannot always be used to resolve which of the sensors are associated with erroneous readings. One such example is during a step change in the sensor's readings in Figure 5. At the point of step change, there is a sudden increase in *x_gr_*(*t*). For the samples that proceed the change point, however, the *x_gr_*(*t*) will become relatively small and fail to indicate which of the sensor's is responsible for the increase in *x_sr_*(*t*). In this case, the erroneous sensor can only be identified by keeping track of its past quality states. This is achieved by conditioning *x_sr_*(*t*) upon the quality state of the previous sample. The CPD in node *G* was parameterized as a Gaussian distribution of the error *x_sr_*(*t*) conditioned upon the current and previous quality states and the gradient test *p*(*x_sr_*(*t*)|*q*(*t*), *q*(*t* − 1), *x_gr_*(*t*)).

### 6.4. Network Training

Each of the four sensors had a DBN trained in a supervised manner using their own set of historical measurements between 19 February 2008 and 8 November 2010 in Sullivans Cove. The training set was comprised of four blocks of sensor observations with 10 minutes sample intervals that were separated in time (a total of 53,886 observations). Moreover, corresponding samples from the co-situated, equivalent sensor at alternate depth and SHOC model hindcasts were used. The model hindcasts were interpolated (with 3 times oversampling) to align the hindcasts with the time stamp of the sensors. The starting date of each observation block occurred after sensors had been cleaned and re-calibrated ensuring the CPD *p*(*q*(*t*)|*q*(*t* − 1), *cl*(*t*), *cal*(*t*)) captured periods of measurement reliability. The training sets were quality assessed by two experts according to the IOC quality scheme detailed in Table 1. The experts had 3 years and 4 years experience in the quality assessment of physical observations from marine sensors and had several years experience studying the hydrodynamic behaviour of Sullivans Cove where the sensors were situated. Models were trained and then tested using the Bayes Network Toolbox [43] in Matlab. The toolbox was modified to incorporate the MAP estimates for the Gaussian and non-uniform Dirichlet priors used in the models.

#### 6.4.1. Prior Distributions

One of the advantages of Bayesian Networks (BN) is that under the assumption that the training data is complete, the BN is modular and each CPD in the network can be trained independently. This training can be performed with or without a prior belief distribution for each CPD. In our proposed quality assessment network, the multinomial CPD of node *C* was provided with a prior distribution to encapsulate our current understanding of the degradation of the EC-1500 sensor in shallow water. Prior belief of the CPD in node *E*, the gradient conditioned upon the quality state, was also provided to encode our understanding of realistic changes in temperature and conductivity observations over short periods of time. The parameters of the two CPDs were learnt from the training set *X* as the maximum a posteriori (MAP):

(6) $$p{\left(\Theta |X\right)}_{\text{max}}=\arg{\text{max}}_{\Theta }\prod_{t=1:N}\prod_{r=1:e}p(X_{t}|\Theta_{r})\cdot p(\Theta_{r})$$

where *p*(Θ|*X*) was the posterior distribution of the parameters Θ of the CPD given e independent vectors of its parental configurations and *N* training examples in *X_t_*. The prior distribution (*p*(Θ)) in node *C* was encoded as a Dirichlet distribution, which is the conjugate of the multinomial distribution [34]. Consequently the MAP could be computed in closed form:

(7) $$\begin{array}{cc} p{\left(\Theta_{p(q(t)|cl(t),\text{cal}(t),q(t-1)})|X\right)}_{\text{max}} & =\frac{N_{z,r}+\tau_{z,r}-1}{N_{r}+\tau_{r}-k}\\ N_{r} & =\sum_{z=1:4}N_{z,r}\text{and}\tau_{r}=\sum_{z=1:4}\tau_{z,r}\text{and}r=1:288 \end{array}$$

where *N_z,r_* is the number of training observations and *τ_z,r_* is the Dirichlet hyper-parameter for the *z^th^* state of sensor quality and the *r^th^* parental configuration of the CPD. Equation (7) shows that larger *τ_z,r_* will have more influence upon the configuration. Consequently, *τ_z,r_* were set to relatively large values between 1 and 100 to ensure they had at least some influence upon learning based upon our past experience of bio-fouling and drift during deployment of EC-1500 sensors. The prior distribution of node *E* was normally distributed. This conjugate prior enabled the MAP to be computed in closed form:

(8) $$p{\left(\Theta_{p(x_{gr}(t)|q(t))}|X\right)}_{\text{max}}=\frac{N_{r}\cdot \sigma_{o,r}}{N_{r}\cdot \sigma_{o,r}+\sigma_{x,r}}\cdot \mu_{x,r}+\frac{\sigma_{o}}{N_{r}\cdot \sigma_{o,r}+\sigma_{o,r}}\cdot \mu_{o,r}$$

where *r* = 1 : 4 are the parental configurations, *μ _o,r_*, *σ_o,r_* is the mean and standard deviation of the prior distribution and $\left(\mu_{x,r},\sigma_{x,r}^{2}\right)$ is the mean and standard deviation of the *x_gr_*(*t*) distribution. The prior distributions were set to *μ_o,r_*_=1:4_ = [0.05 0.3 0.7 4], *σ_o,r_*_=1:4_ = [0.1 0.2 0.3 5] for the two temperature networks and *μ_o,r=_*_1:4_ = [250 1300 2300 4500], *σ_o_,_r_*_=1:4_ = [150 600 1000 2000] for the two conductivity networks. Prior distributions were non-informative for the remaining CPDs of the DBN, and hence, these CPDs were parameterized by maximizing the likelihood *p*(*X*|Θ*_N_*)*_max_*.

### 6.5. Quality State Assessments

After the network was trained, the operational phase of quality assessment proceeded. As each sensor reading streamed into the information system, a quality state inference was performed upon its corresponding DBN:

(9) $$\begin{array}{cc} p(q(t)|e(1:t)) & =\frac{p(q(t),e(1:t))}{\sum_{r=1}^{4}p(qr(t),e(1:t))}\\ \text{where}\;p(q(t),e(1:t)) & =p(\text{cal}(t))\cdot p(cl(t))\cdot p(s(t))\cdot p(q(t)|q(t‐1),\text{cal}(t),cl(t))\cdot p(x_{sr}(t)|q(t),q(t‐1),x_{gr}(t))\cdot p(x_{gr}(t)|q(t))\cdot p(x_{mr}(t)|q(t))\cdot p(x_{sd}(t)|q(t),s(t)) \end{array}$$

given e(1 : *t)* represent the observed causes, effects and states of the network to the current sample.

### 6.6. Generation of Uncertainty Measurements

Once probabilistic assessments of the IOC flags were obtained, they were then used to generate a quantitative assessment of each measurement's uncertainty. A continuous quality metric was generated using a similar approach to the fuzzy set approach [14] with contributions from the posterior probabilities as opposed to fuzzy memberships. These probabilistic estimates of the DBN quality states were mapped to a continuous quality metric *eb*(*t*):

(10) $$eb(t)=\sum_{r=1}^{4}w_{r}\cdot p(q_{r}(t)|e(1:t))$$

where *w_r_* are the quality state weights for the conductivity and temperature sensors shown in Table 2. The weight (*w*_1_) associated with the the good flag was assigned with the values of the EC-1500 sensor accuracy quoted by the manufacturer. The other weight values were based upon the empirical values computed in [14] but modified to incorporate 4 quality states instead of 3 states.

## 7. Results and Discussion

Whilst the end goal of the proposed quality assessment system is to generate uncertainty measures of samples, we first investigate the accuracy of the quality state assessments of the DBN. The DBN of each sensor was compared to its corresponding static BN using the same test and training data sets. The structure of the static BN is shown for a shallow temperature sensor in Sullivans Cove in Figure 1. The static BN exploits the same suite of cause and observed evidence variables that were described for the DBN (Figure 3) in Section 6. The only difference between the networks being the co-situated sensor and quality state nodes for the current sample of the DBN are dependent upon the previous sample's quality state. A test set was prepared for each sensor consisting of a segment of 19,375 observations sampled at 10 minute intervals between 1 September 2011 and 1 December 2011. This test set was separate from the training set. Each test set was also comprised of the corresponding observations of the equivalent sensor at an alternate depth and SHOC model hindcasts (for the shallow sensors).

### 7.1. Quality Assessment Metrics

A discrete quality metric of the DBN and static BN was obtained for each sample by computing the distance between the quality state with maximum posterior probability and the expert selected quality state. In addition, a continuous quality label (*cq*(*t*)) was computed from the quality state posteriors of each network. The distance between the continuous label and corresponding manually assessed quality label was used as a performance metric:

(11) $$\begin{array}{cc} d(t) & =|m(t)-cq(t)|\\ cq(t) & =\sum_{r=1}^{4}w_{r}\cdot p(q_{r}(t)|e(1:t)) \end{array}$$

where *w_r_* = [1 2 3 4] is the weight vector composed of the quality states.

### 7.2. Quality Assessment Results

**Figure 6.**
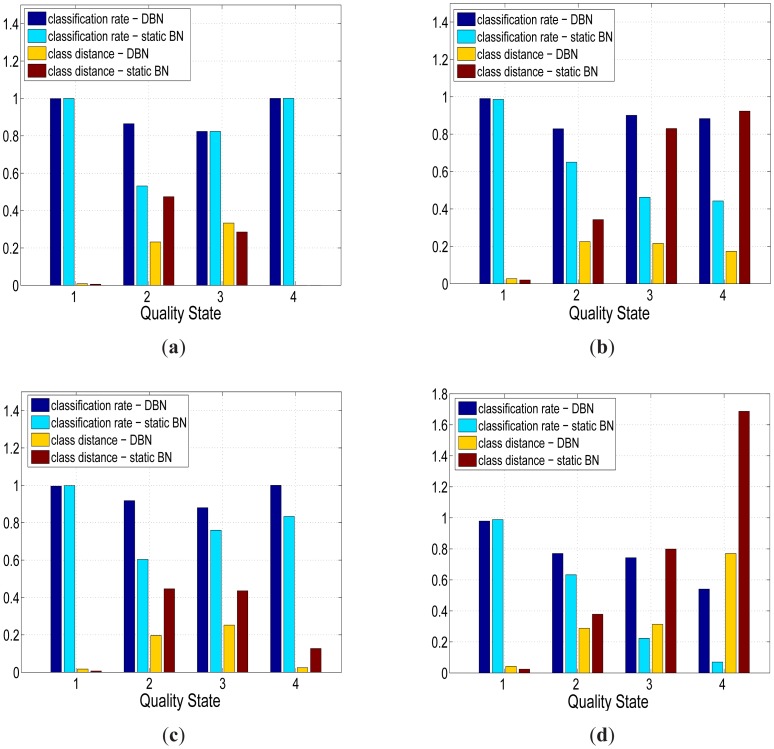
The automated quality assessments from two BN (static BN and DBN) compared to expert quality assessments across the four sensors in Sullivans Cove, Hobart. The quality state labels 1 to 4 define the quality states in Table 1. There were two metrics used to compare the assessment performance of the Bayesian networks. The first metric was the distance between the quality state with maximum posterior probability and the quality state selected by an expert. The second metric was the distance between the expert's quality state and the continuous quality label of the network defined in Equation (11). (**a**) Temperature Sensor at 1 m; (**b**) Conductivity Sensor at 1 m; (**c**) Temperature Sensor at 10 m; (**d**) Conductivity Sensor at 10 m.

Figure 6 shows that the quality assessments generated by the DBN and static BN were in close agreement with the expert based quality assessments for each of the four sensors. The average quality assessment accuracy of the temperature sensors (Figure 6(a),6(c)) was higher than the conductivity sensors (Figure 6(b),6(d)) with an improvement of 1.22%, 11.4%, 3.65% and 40% for the good, probably good, bad but potentially correctable (to be referred to as probably bad from this point) and bad quality states respectively. The average quality assessment accuracy for the good class of sensor data was 99.1%, which was consistently higher than the remaining quality classes. The average assessment accuracy was lowest for the probably bad class at 83.7 % although still relatively close to the average accuracy of the probably good quality state of 84.6% and bad quality state of 85.6%.

The lower assessment accuracy of the “degraded” quality states could be associated with the natural imbalance in the training data set where the good quality state had far more examples to use in order to learn the network than the “degraded” states. Furthermore, the “degraded” states had far greater overlap between their boundaries. This is because experts found it easier to label the samples as good; the difficulty in assessing with the IOC quality scheme was labeling “degraded” samples in a consistent manner across an entire data set *i.e.*, similar samples may be labeled as probably good in some instances and probably bad in other instances.

**Figure 7.**
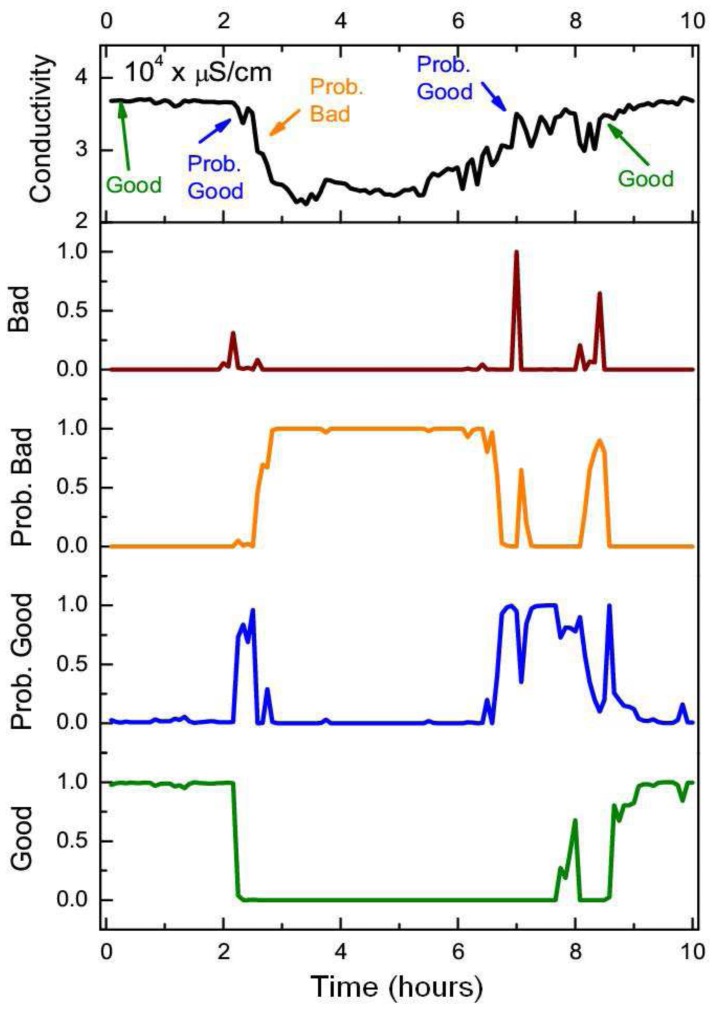
The top time-series of each figure corresponds to a section of sensor readings from the conductivity sensor at 1 m that have been assessed by a human expert and automatically flagged by the DBN. The four bottom series correspond to the posterior probabilities of the (between 0–1) quality states of each sensor sample in the network. The arrows upon the top time-series correspond to the points at which the expert's assessment has changed to the quality state specified.

**Figure 8.**
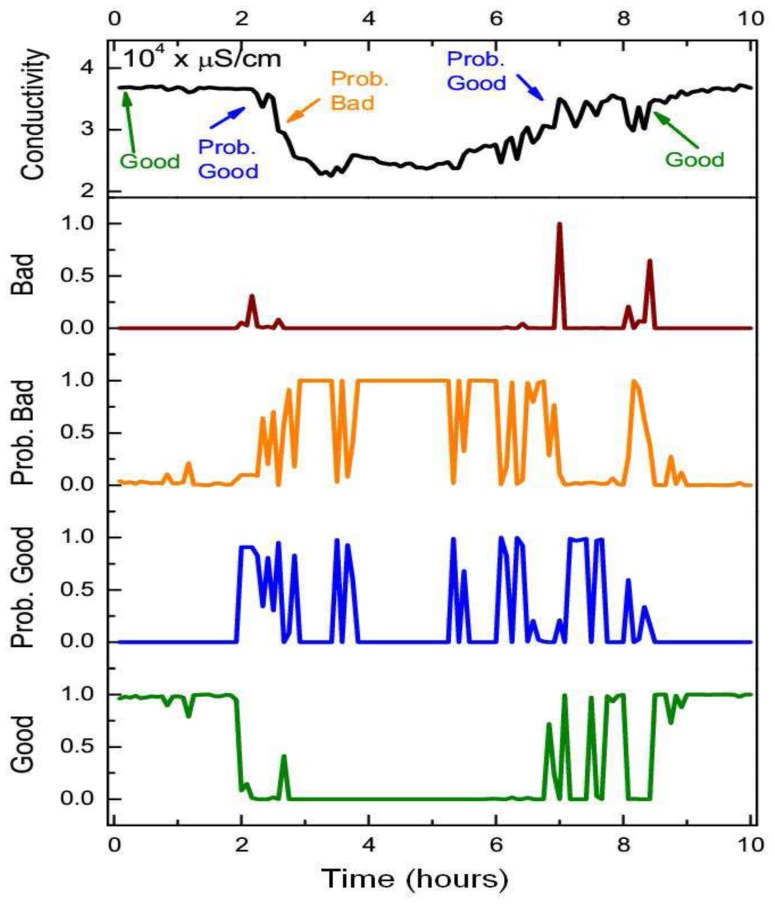
The top time-series of each figure corresponds to a section of sensor readings from the conductivity sensor at 1 m that have been assessed by a human expert and automatically flagged by the static BN. The four bottom series correspond to the posterior probabilities of the (between 0–1) quality states of each sensor sample in the network. The arrows upon the top time-series correspond to the points at which the expert's assessment has changed to the quality state specified.

Whilst the static BN showed a slightly higher average quality assessment accuracy across the good quality state of samples (0.22%), the DBN offered a statistically significant improvement (two sample t-test at *p* = 0.05) in assessment performance for each of the “degraded” quality states. The DBN offered an average improvement of 41%, 46.6% and 33.7% for the probably good, probably bad and bad states respectively. The overall quality assessment accuracy of the DBN was still greater than the static BN despite the heavy imbalance towards the good quality state in the training set, which was comprised of 96% of the observations. Whilst the DBN mislabeled an additional 123 samples from the good state compared to the static BN, the DBN correctly labeled an additional 1,292 samples across all three “degraded” quality states. The distance metric (defined in Equation (11)) mirrored the previous improvement of the DBN with an average quality state distance that was 41%, 40% and 54.8% lower than the static network across the same three “degraded” quality states. The improved quality assessment of the DBN could be attributed to the greater consistency in labeling data segments with constant quality states. One negative aspect of this labeling consistency was that the DBN tend to label the start and end positions of erroneous segments with less accuracy than the static BN. This was one of the reasons why the static BN offered a slightly higher assessment accuracy than the DBN for the good quality state. This small drop in assessment accuracy, however, was outweighed by the DBN's improvement in assessing the segments of erroneous sensor readings. The DBN was shown to better replicate expert assessment than the static BN across a series of segments with constant quality states in Figure 7. In Figure 8, the quality assessments of the static BN tend to jump between the good, probably good and probably bad states more frequently than the DBN, which label homogeneous segments of data quality with greater consistency.

### 7.3. Uncertainty Measures

A 2,672 sample subsection of the test-set was manually assessed by one of the experts in order to place error bars upon the sensor readings. This test-set was identical to the one used to evaluate the quality assessment performance of the fuzzy logic system in [14]. The uncertainty measure of samples were calculated from the quality state posteriors of the DBN and static BN using Equation (10). The algorithm performance was measured by expressing the data uncertainty as a percentage of the manually computed error bars. The test set percentages were presented as histogram tables for the temperature (Table 3) and conductivity sensors (Table 4) to enable direct comparison with the results of the fuzzy logic system reported in [14]. Most of the tests utilised in the fuzzy system [14] were identical to Section 6. One variation was the test associated with the difference between co-situated sensors. In the fuzzy system this was formulated as the difference between sensor values as opposed to the difference in the linear regression model in Section 6.3.4. Furthermore, an additional test was utilized in the shallow DBN at 1 m to model the difference between the sensor readings and SHOC model hindcasts (Section 6.3.3).

The results from Table 4 indicate that the DBN produced data quality uncertainty measures that fell within an acceptable range of performance (ratio of 66%–150%) for an average of 78.7% of the conductivity test sets. The correspondence between the automatic uncertainty measure and manually generated error bars was shown to be higher for the temperature sensors in Table 3 achieving an acceptable range of performance for an average of 95.1% of the test set. A comparison of the DBN and static BN quality measures showed that the DBN offered a statistically significant improvement in replicating the manual error bars in three of the four sensor test sets (two sample t-test at *p* = 0.05). This improvement was a result of the higher overall quality assessment performance of the DBN as described in Section 7.1. It was discovered, however, that higher assessment accuracy did not guarantee closer agreement with the error bars. The test-set associated with the conductivity sensor at 10 m (Table 4(b)) is one such example. Such outlier examples are the result of manual generation of error bars with a far more stochastic process than the linear mapping of class posteriors.

A comparison of the DBN and fuzzy system shows that the automated uncertainty measure of the DBN replicate the manual error bars with far greater accuracy than the fuzzy system. The DBN produced an average improvement of 15% across both temperature sensors (Table 3) and an average improvement of 53% across both conductivity sensors (Table 4). The main difference between the two systems was that the fuzzy logic system was parameterized using prior knowledge exclusively, whilst the DBN system was parameterized with prior knowledge and data sets representing the local context. The fuzzy logic system relies upon a set of expert selected parameters that are potentially biased and may not be representative of the local context.

## 8. Discussion

One of the underlying assumptions of automated quality assessment is that systems require historical observations to either learn the normal behavior of the sensor (in outlier detection) or parameterize specific tests. This is an issue for recently deployed sensors that have not built up a sufficient observational history. In these cases, the only available option is to exploit prior knowledge. This could take the form of an expert's knowledge of the observed phenomena and/or an independent data set that is representative of the deployment region. Such options are likely to introduce bias into the initial assessment systems, and consequently, it is important that such systems can be updated with the arrival of new data. This requires the new data to be manually assessed to update the DBN. Consequently, the update cannot be conducted in real-time and the quantity of newly assessed samples must be limited, if the approach is to remain practical. A strategy to select the most influential samples must be developed; these could be samples with high classification uncertainty (*i.e.*, samples at the boundary of multiple classes) or samples that were assessed as erroneous. This will be addressed in future work.

Another critical issue in automatic quality assessment is that it is difficult to distinguish between sensor errors and unusual events. This is of particular concern with a sparse distribution of sensors where unusual events can potentially be flagged as bad data. In the case of environmental monitoring, it is often these unusual events that are of particular interest. Therefore, although QA/QC can build confidence in the data provided, we want to be sure that interesting events are not ignored, or filtered out, and that effort is not wasted in servicing sensors that may appear to be faulty but are working correctly. An approach has been developed to address this problem for TasMAN using an autonomous vehicle and real-time data to provide the context to differentiate between an error and an event [44]. Sensors reporting bad data are identified using automated quality control, which the vehicle can access with a publicly available web service along with the real-time sensor readings. If the data is consistently flagged as bad and there are no public sources of evidence to support an environmental event is occurring, the autonomous vehicle visits the sensor nodes so that it might compare its onboard temperature and salinity sensors with those of the sensor reporting data of uncertain quality. If there is then a significant difference between the readings, a recommendation is made for the sensor to be serviced *i.e.*, cleaned and/or calibrated.

## 9. Conclusions

A Bayesian Network (BN) based framework was proposed to provide automated quality assessments of streamed sensor data. The framework models the causal relationship between variables of the quality assessment process without being constrained by the restrictive assumptions that outlier detection or classification approaches may place upon practical deployments. The original BN framework is modified from treating each sample independently (the static BN) to exploiting the sequential correlation of readings using a Dynamic Bayesian Network (DBN). A comparison of the DBN and static BN algorithms implemented upon sensors in Sullivans Cove, Hobart indicate the DBN better replicates the quality assessments provided by experts. In particular, the classification accuracy of the “degraded” quality classes were superior as the DBN managed to classify segments of erroneous readings with greater accuracy than the static BN. The posterior estimates of the quality states were mapped to a more meaningful measure of sample uncertainty. The uncertainty measure was compared to a similar measure derived using a fuzzy logic approach [14]. The BN approaches were shown to offer a substantial average improvement of 15% and 53% in replicating the error bars of experts for the temperature and conductivity sensors respectively.

## Figures and Tables

**Table 1.** The four quality states specified for the quality flag scheme of the Intergovernmental Oceanographic Commission (IOC). These four discrete states are used to represent the hidden quality state (node C in Figure 3) of the Bayesian network implementation. Each quality assessment involves performing an inference upon these quality states given the observed cause and effect variables of the network. The quality state of code 3 “bad data that is potentially correctable” was referred to as probably bad data in the experiment.

**Quality Code**	**Code Meaning**
1	Good data
2	Probably good data
3	Bad data that are potentially correctable (Probably bad data)
4	Bad data

**Table 2.** The weights used to generate error bars for each assessed data sample based upon the posteriors of the quality states. The weight w_1_ corresponds to the sensor accuracies specified by the manufacturer whilst the other values were based upon the empirical values in [14] but modified to incorporate 4 classes as opposed to 3 classes.

**Sensed Phenomenon**	*w*_1_ **- Good**	*w*_2_ **- Prob Good**	*w*_3_ **- Prob Bad**	*w*_4_ **- Bad**
Conductivity	0.1	0.3	1.2	3
Temperature	0.2	1	4	8

**Table 3.** A comparison of uncertainty measures of the DBN (section 6), static BN [15] and fuzzy logic assessment systems [14] expressed as a histogram of the ratio of automatic quality metrics and manual error bars across a test-set of 2,672 samples for the temperature sensors in Sullivans Cove. (**a**) Temperature sensor situated at a depth of 1 m; (**b**) Temperature sensor situated at a depth of 10 m.

**Automatic EB / Manual EB**	**DBN**		**static BN**		**fuzzy logic**	
	**instances**	%	**instances**	%	**instances**	%
Above 300 %	13	0.5	16	0.6	13	0.5
200–300 %	57	2.1	55	2.1	166	6.2
150–200 %	36	1.4	117	4.4	119	4.5
66–150 %	2,488	93.1	2,374	88.9	2,131	79.8
50–66 %	42	1.6	56	2.1	108	4.0
33–50 %	28	1.0	36	1.4	92	3.4
Less than 33%	8	0.3	18	0.6	43	1.6

(**a**)						

**Automatic EB / Manual EB**	**DBN**		**static BN**		**fuzzy logic**	
	**instances**	%	**instances**	%	**instances**	%
Above 300 %	9	0.3	8	0.3	1	0.0
200–300 %	27	1.0	50	1.9	339	12.7
150–200 %	11	0.4	113	4.2	84	3.1
66–150 %	2,592	97.0	2421	90.6	2,224	83.2
50–66 %	24	0.9	52	1.95	9	0.3
33–50 %	6	0.2	14	0.5	7	0.3
Less than 33%	3	0.1	14	0.5	8	0.3

(**b**)						

**Table 4.** A comparison of the uncertainty measures of the DBN (section 6), static BN [15] and fuzzy logic assessment systems [14] expressed as a histogram of the ratio between the automatic quality metrics and manual error bars across a test-set of 2,672 samples for the conductivity sensors in Sullivans Cove. (**a**) Conductivity sensor situated at a depth of 1 m; (**b**) Conductivity sensor situated at a depth of 10 m.

**Automatic EB / Manual EB**	**DBN**		**static BN**		**fuzzy logic**	
	**instances**	%	**instances**	%	**instances**	%
Above 300 %	0	0.0	14	0.5	157	5.9
200–300 %	154	5.8	112	4.2	140	5.2
150–200 %	226	8.5	139	5.2	138	5.2
66–150 %	2,054	76.9	1,802	67.4	993	37.2
50–66 %	131	4.9	268	10.0	342	12.8
33–50 %	86	3.2	204	7.6	365	13.7
Less than 33%	21	0.8	133	5.0	537	20.1
(**a**)							

**Automatic EB / Manual EB**	**DBN**		**static BN**		**fuzzy logic**	
	**instances**	%	**instances**	%	**instances**	%
Above 300 %	92	3.4	91	3.4	265	9.9
200–300 %	18	0.7	94	3.5	170	6.4
150–200 %	27	1.1	116	4.3	99	3.7
66–150 %	2,151	80.5	2,208	82.6	980	36.7
50–66 %	128	4.8	83	3.1	336	12.6
33–50 %	123	4.6	62	2.3	268	10.0
Less than 33%	133	5.0	18	0.7	547	20.5
(**b**)							
